# Exploration of Molecular Mechanisms of Immunity in the Pacific Oyster (*Crassostrea gigas*) in Response to *Vibrio alginolyticus* Invasion

**DOI:** 10.3390/ani14111707

**Published:** 2024-06-06

**Authors:** Enshuo Zhang, Zan Li, Luyao Dong, Yanwei Feng, Guohua Sun, Xiaohui Xu, Zhongping Wang, Cuiju Cui, Weijun Wang, Jianmin Yang

**Affiliations:** 1 School of Agriculture, Ludong University, Yantai 264025, Chinalizanlxm@163.com (Z.L.); xxh83121@163.com (X.X.); cuicuiju@163.com (C.C.); 2 Yantai Haiyu Marine Technology Co., Ltd., Yantai 264000, China; 3 College of Fisheries and Life Science, Shanghai Ocean University, Shanghai 201306, China; 4 Yantai Kongtong Island Industrial Co., Ltd., Yantai 264000, China

**Keywords:** bivalve, pathogen infection, gill, RNA sequencing, protein–protein interaction networks, hub genes

## Abstract

**Simple Summary:**

As a filter-feeding and sessile invertebrate living in estuaries and intertidal areas, *Crassostrea gigas* must cope with and adapt to a dynamic and changeable environment. *Vibrio alginolyticus* is a Gram-negative bacterium that is widespread in oceans and estuaries, and is one of the main *Vibrio* species that cause oyster disease. In this study, we used transcriptome sequencing to help us better understand how the giant oyster adapts to pathogen-rich environments. By focusing on the gills, which play a crucial role in the immune response, we aimed to shed light on the molecular processes underlying the interaction between the oyster and the pathogen.

**Abstract:**

Over the years, oysters have faced recurring mass mortality issues during the summer breeding season, with *Vibrio* infection emerging as a significant contributing factor. Tubules of gill filaments were confirmed to be in the hematopoietic position in *Crassostrea gigas*, which produce hemocytes with immune defense capabilities. Additionally, the epithelial cells of oyster gills produce immune effectors to defend against pathogens. In light of this, we performed a transcriptome analysis of gill tissues obtained from *C. gigas* infected with *Vibrio alginolyticus* for 12 h and 48 h. Through this analysis, we identified 1024 differentially expressed genes (DEGs) at 12 h post-injection and 1079 DEGs at 48 h post-injection. Enrichment analysis of these DEGs revealed a significant association with immune-related Gene Ontology (GO) terms and Kyoto Encyclopedia of Genes and Genomes (KEGG) pathways. To further investigate the immune response, we constructed a protein–protein interaction (PPI) network using the DEGs enriched in immune-associated KEGG pathways. This network provided insights into the interactions and relationships among these genes, shedding light on the underlying mechanisms of the innate immune defense mechanism in oyster gills. To ensure the accuracy of our findings, we validated 16 key genes using quantitative RT-PCR. Overall, this study represents the first exploration of the innate immune defense mechanism in oyster gills using a PPI network approach. The findings provide valuable insights for future research on oyster pathogen control and the development of oysters with enhanced antimicrobial resistance.

## 1. Introduction

The Pacific oyster (*Crassostrea gigas*) is among the most important aquatic shellfish farming species in the world and has a commercially important economic value [1,2]. Oysters, scallops and other shellfish lack specific humoral immunity or cellular immunity mediated by various lymphocytes, and can only rely on innate immunity to adapt to the dynamically changing external environment [3]. However, *C. gigas* generally live year-round in estuarine and intertidal waters where the environment is complex and pathogen-rich [4]. It is a question to be explored how oysters lacking specific immunity can survive in a changing and complex environment. Although the oyster is an invertebrate, evidence is mounting that the oyster has developed a highly sophisticated immune system that regulates itself in response to external pathogens and environmental stresses [5,6,7,8].

For many years, oysters have frequently experienced mass mortality during the summer breeding season [9]. There are many reasons why summer is the main season for oyster mortality, such as viral and bacterial infections and increased water temperatures [10]. Many oyster mortalities during culture are attributed to *Vibrio*. The pathogenic *Vibrio*s that have been reported include *Vibrio alginolyticus*, *Vibrio splendens*, and *Vibrio giganteus* [11]. *V. alginolyticus* is a widely distributed marine Gram-negative bacteria, which causes oyster disease and even mortality of shellfish and shrimp, resulting in severe economic loss [12,13,14]. *V. alginolyticus* infection in humans has been reported to cause wound infections [15] and ear infections [16]. For bivalves, the gills are the first organ to contact the external environment [17]. Tubules of gill filaments were confirmed to be in the hematopoietic position in *C. gigas*, which produce hemocytes with immune defense capabilities [18]. At the same time, the epithelial cells of oyster gills produce immune effectors to defend against pathogens [19]. Thus, the transcriptome sequencing of oyster gill tissue helps us to better understand how the oyster adapts to pathogen-rich environments.

In recent years, the advancement of high-throughput transcriptome sequencing technology has enabled the comprehensive analysis of mRNA expression in various tissues and organs. This technique has been extensively applied in bivalves, including *Crassostrea angulata*, *Pinctada fucata*, and *Crassostrea virginica* [20,21,22]. It is possible to gain insights into the intracellular regulatory network and molecular mechanisms by performing the transcriptome sequencing of specific tissues or organs [23]. Therefore, applying this approach to study the immune regulation mechanisms in oyster gill tissue following infection with *V. alginolyticus* can provide valuable insights into host–pathogen interaction.

In this research, we investigated the immune mechanism of oysters after 12 and 48 h of injection with *V. alginolyticus* by transcriptomics and protein–protein interaction (PPI). The main focus is on the screening of DEGs, GO and KEGG functional enrichment analysis. Subsequently, we produced a PPI network based on the KEGG pathway results in order to obtain the key gene. Lastly, we screened to obtain 16 key genes based on the KEGG pathway and PPI results, and identified them using real-time quantitative PCR (RT qPCR). Summarily, the findings of this study provide valuable resources for understanding the immune mechanisms of *C. gigas* during *V. alginolyticus* infection, thus contributing to the development of greater disease resistance in oysters.

## 2. Materials and Methods

### 2.1. Crassostrea gigas and Vibrio alginolyticus

The oysters used in this study were sourced from Kongtong Island in Yantai, Shandong Province, China. These oysters were approximately six months old and their weight and shell length were 26.59 ± 7.00 g (mean ± SD) and 63.35 ± 4.73 mm (mean ± SD). They were temporarily housed in six glass tanks, with 30 oysters per tank. The tanks were replaced daily with filtered seawater and the oysters were fed with spirulina as their diet. For the experimental injection, we utilized *V. alginolyticus* obtained from the research team led by Changming Bai [24]. Prior to injection, the *V. alginolyticus* bacteria were cultured for 10 h at 28 °C in 2216 E liquid medium. To ensure accurate sampling, the oysters were suspended from feeding with spirulina for two days before the sampling.

### 2.2. Sample Treatment and RNA Extraction

In this experiment, we designed three different treatment groups: the blank control group (BCG), the PBS control group (PCG), and the *V. alginolyticus* experiment group (VEG). The BCG served as a control with no treatment applied. At the beginning of sampling, gill tissues of oysters were placed in liquid nitrogen and then transferred to a −80 °C refrigerator to prepare for sequencing. For the PCG and VEG, 50 μL of PBS and 50 μL of *V. alginolyticus* (2 × 10^9^ CFU) were injected into the adductor muscle of oysters at the beginning of the experiment. Then, the gills of PCG and VEG oysters were collected in the same manner as the BCG at 12 h and 48 h post-injection. For each treatment group at different time points, we randomly selected six oysters to extract RNA from the gills: BCG at 0 h (BCG-0 h), PCG at 12 h (PCG-12 h), VEG at 12 h (VEG-12 h), PCG at 48 h (PCG-48 h), and VEG at 48 h (VEG-48 h). At each sampling time, RNA from two oysters was mixed in the same ratios to set up three sets of biological replicates for the construction of RNA libraries.

### 2.3. Library Construction and RNA Sequencing

The extraction of total RNA from the oyster gill tissue was performed using TRIzol reagent (Invitrogen, Waltham, MA, USA) following the manufacturer’s method. The quality of RNA is shown in Table S1. The extracted RNA was then divided into two portions. One portion was used for library construction, while the remaining RNA was reserved for qPCR validation. The library construction process was carried out based on the methods previously described [25,26]. To obtain sequencing data, the constructed libraries were subjected to sequencing using the Illumina Novaseq platform. The sequencing process generated 150 bp paired-end reads, providing high-quality data for subsequent analysis.

### 2.4. Processing of Data and Differential Expression Analysis

To ensure the accuracy of our subsequent analyses, we performed a filtering step on the raw data. This involved removing readings that contained adapters, poly-N sequences, and low-quality sequences. Additionally, we assessed the quality of the clean data by calculating metrics such as Q20, Q30, and GC content. In order to obtain accurate gene expression levels, FPKM was applied to eliminate the effects of sequencing depth and gene length on reads. For the differential expression analysis, we used the DESeq2 software (version 1.20.0) (http://www.bioconductor.org/packages/release/bioc/html/DESeq2.html (accessed on 28 September 2023)). We applied a filtering criterion of a *p*-value ≤ 0.05 and |log_2_ fold change| of ≥ 1 to select DEGs.

### 2.5. Functional Enrichment Analysis and PPI Networks

To gain a better understanding of the biological functions and pathways associated with the DEGs, we performed GO and KEGG enrichment analyses. These analyses were conducted using the DAVID v6.8 software (https://david.ncifcrf.gov (accessed on 10 October 2023)).

The immune-related genes enriched in the KEGG pathway were used to build a PPI interaction network through the online tool STRING V11.5 (https://cn.string-db.org (accessed on 10 October 2023)) to investigate linkages and interactions of genes involved in the immunity of oysters.

### 2.6. Gene Expression Validation by RT-PCR

To validate the accuracy of RNA sequencing results, we validated a total of 16 DEGs using qPCR. This specific primer for 16 genes related to immunity was designed using Primer Premier 5.0 (PREMIER Biosoft, San Francisco, CA, USA) (Table S2). The stability verification of five housekeeping gene candidates followed the approach of our previous study [27]. Finally, *EF-1α* with the best stability was selected as the housekeeping gene. The reaction mix and profile of RT qPCR followed those set out in our previous study [28]. We used the 2^−ΔΔCT^ method to calculate the relative expressions of 16 genes.

### 2.7. Histological Analysis

Prior to transcriptome sequencing, we conducted histological examinations of oyster gill tissue. Gill samples were fixed in 4% PFA solution for 24 h and dehydrated in different concentrations of methanol. Subsequently, the tissue was made transparent with xylene, embedded in paraffin, sectioned, and stained with hematoxylin and eosin. Finally, a light microscope (Nikon (Minato City, Tokyo, Japan), DS-Fi2) was used to observe the samples.

## 3. Results

### 3.1. Identification of DEGs

Transcriptome sequencing was performed for BCG-0 h, PCG-12 h, VEG-12 h, PCG-48 h and VEG-48 h, with three biological replicates for each group. The correlation coefficients between the transcriptome data of the three biological replicates are shown in Figure S1. The obtained reads were filtered and mapped to the *C. gigas* reference genome [29]. Approximately 25,000 genes (FPKM > 0) were detected on average in each of the five groups (Table S3). To gain functional insights, all identified genes were compared with the SwissProt database for functional annotation.

### 3.2. Analysis of DEGs

**Figure 1:**
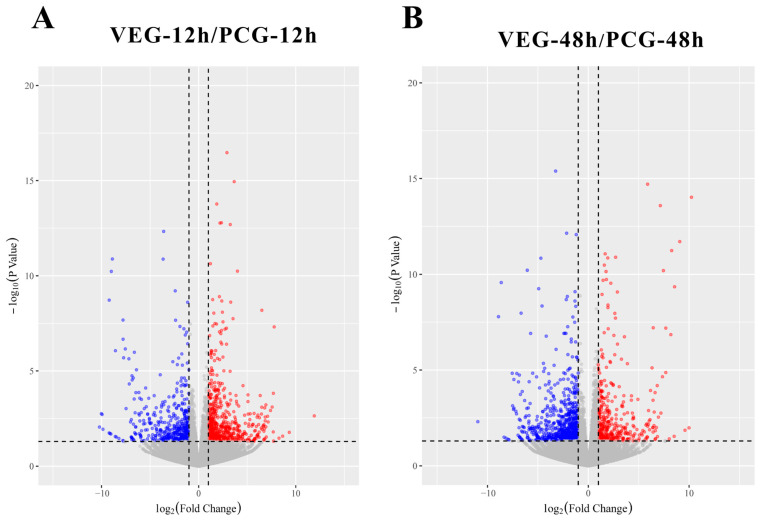
(**A**) Gene expression of VEG-12 h and PCG-12 h samples. Dots in this graph denote the genes. Red dots correspond to DEGs with upregulated expression; blue dots correspond to DEGs with downregulated expression, and gray dots are not DEGs. (**B**) Gene expression of VEG-48 h and PCG-48 h samples.

**Figure 2:**
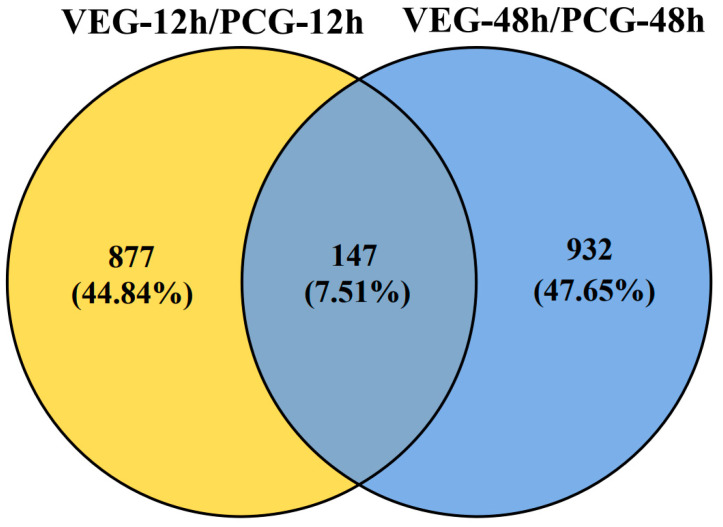
Venn diagram showing the overlapping DEGs at 12 (yellow) and 48 (blue) h post-injection. Here, 877 DEGs are differentially expressed only at 12 h of injection; 932 DEGs are differentially expressed only at 48 h of injection; and 147 DEGs are differentially expressed at both time points.

**Figure 3:**
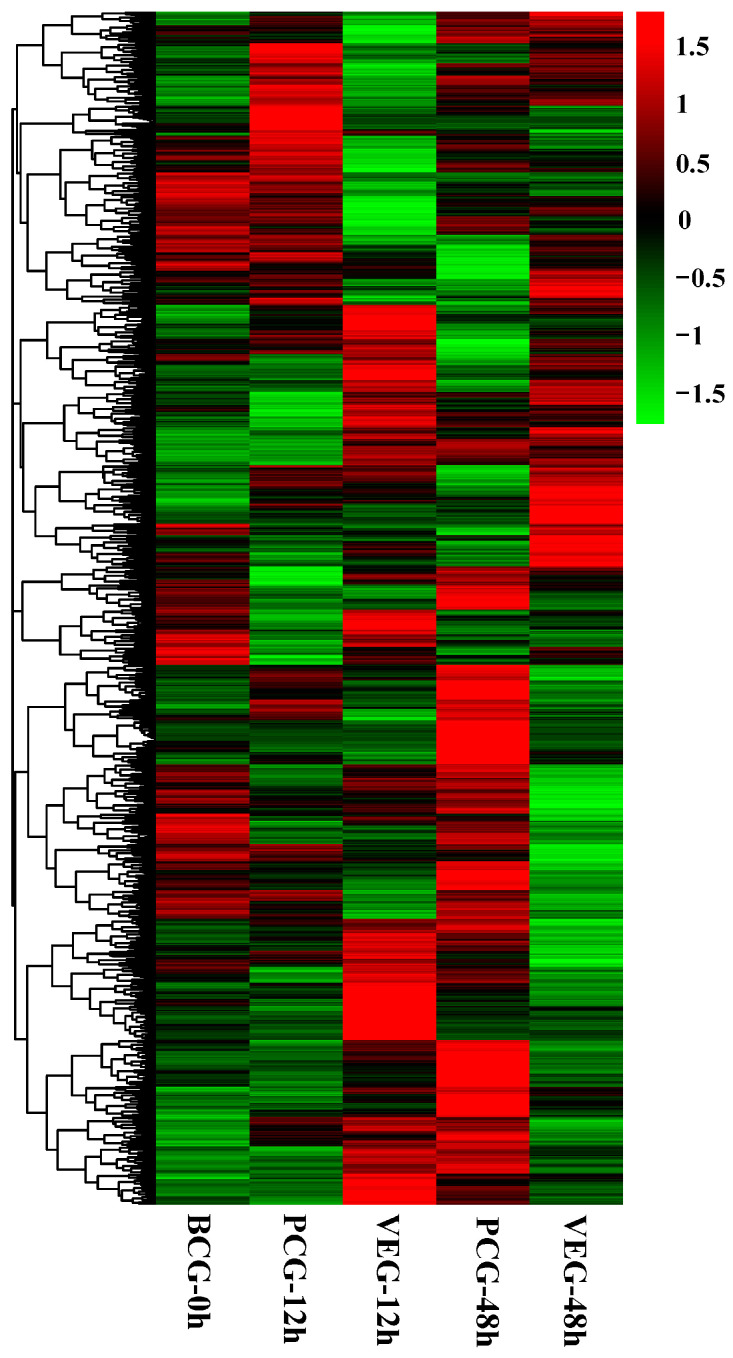
The heatmap of cluster analysis for all DEGs. Expression levels of DEGs are normalized using the log_10_ FPKM method. Each column represents a sample group and each row stands for a DEG. Genes FPKM from low to high are shown in green to red in the graph.

To understand the immune response of *C. gigas* infected with *V. alginolyticus*, we compared VEG-12 h and PCG-12 h as well as VEG-48 h and PCG-48 h, and obtained two sets of DEGs, 1024 and 1079, respectively. Among them, at 12 h post-injection, there were 581 upregulated DEGs and 443 downregulated DEGs, while at 48 h post-injection, 371 DEGs exhibited an upward trend and 708 DEGs displayed a downward trend (Figure 1). The Venn diagram (Figure 2) depicts the overlap of DEGs in oysters infected with *V. alginolyticus*; 877 DEGs were differentially expressed only 12 h after injection; 932 DEGs were differentially expressed only 48 h after injection; and 147 DEGs were differentially expressed at both time points. All of these DEGs may be involved in the immunity of *C. gigas*, so a union of them (1956) was selected for future analysis (Table S4). The clustering heatmap (Figure 3) visually represents the expression patterns and clustering distribution of these DEGs. We can see from the heat map that there are also differences in gene expression patterns between BCG and PCG, which are most likely due to the PBS buffer and injection stimulation, and so we designed the PCG to eliminate these interfering conditions to ensure more accurate experimental results.

### 3.3. GO and KEGG Functional Enrichment Analysis of DEGs

**Figure 4:**
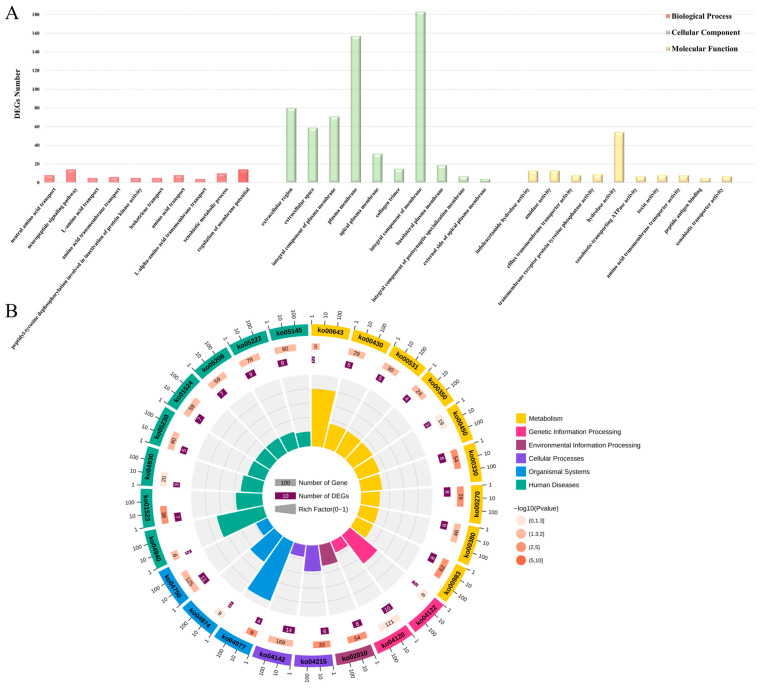
(**A**) GO enrichment analysis of DEGs. The horizontal coordinate stands for the enriched level-3 GO subclasses, and the vertical coordinate stands for the number of DEGs for the corresponding level-3 GO subclasses. (**B**) KEGG enrichment analysis of DEGs. From the outer circle to the inner circle, the first circle represents the level-2 enrichment pathway; the second circle represents the number of background genes and *p* value in the pathway—the more genes there are, the longer the bar is; the third circle represents the number of DEGs in the pathway; the fourth circle represents the Rich Factor value in each pathway.

In our study, we conducted functional enrichment analyses using all 1956 DEGs at 12 and 48 h post-injection. The GO function enrichment analysis identified 80 level-3 biological processes, 23 level-3 cellular components, and 50 level-3 molecular function subclasses. Multiple GO terms play a key role in the response of the *C. gigas* to *V. alginolyticus*, such as positive regulation of inflammatory response, response to bacterium, response to lipopolysaccharide, defense response to Gram-negative bacterium, and so on (Table S5). Figure 4A shows the top 10 level-3 GO terms for biological processes, cellular components, and molecular functions. Furthermore, we performed a KEGG enrichment analysis to gain a deeper understanding of the specific functions of the DEGs, particularly those related to immunity. Specifically, 119 out of 1956 DEGs were enriched to 76 KEGG signaling pathways (Figure 4B). Among them, 12 level-3 KEGG signaling pathways related to immunity, including TRP channel-mediated inflammation regulation, central carbon metabolism in cancer, the PI3K-Akt signaling pathway, and Salmonella infection, were significantly enriched (Table 1).

### 3.4. Analysis of Important DEGs Related to Immune Responses

**Figure 5:**
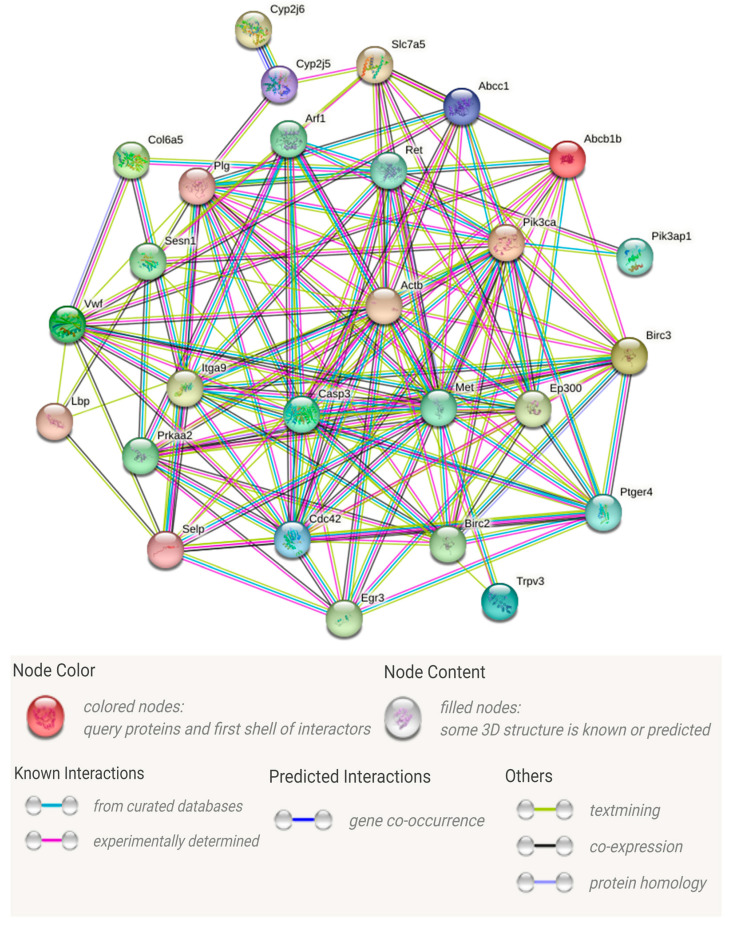
Protein–protein interaction networks. The nodes in the network represent proteins, and the edges represent the interaction relationships between different proteins.

In order to further identify key genes that play important roles in the immune response, we utilized the 35 DEGs in the 12 enriched immune-related signaling pathways (Table 1) to construct the PPI network (Figure 5). Information about specific network parameters is shown in Table 2. From Figure 5 and Table 2, it can be seen that the number of protein interaction edges corresponding to the immune-related DEGs we selected is higher than the expected number of edges, indicating that there is a significant interaction relationship between these genes. Those genes with multiple protein interaction relationships may be hub genes that play important roles in immune regulation. We then identified a total of 16 key immune-related genes with multiple interactions or involved in multiple KEGG signaling pathways based on the analysis of KEGG signaling pathway enrichment and PPI networks (Table 3). *CASP3*, *MET*, and *PIK3CA*, as the genes with the highest numbers of interactions and enriched to multiple pathways, are the most likely to play a key role in the immune regulation of *C. gigas* against *V. alginolyticus*.

### 3.5. Quantitative RT-PCR Validation

**Figure 6:**
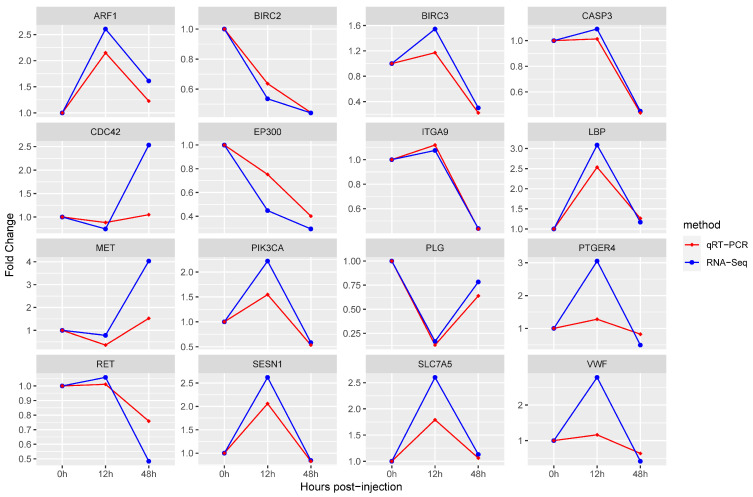
RNA-Seq and qRT-PCR expression trends of 16 key immune-related DEGs. Expression levels of key genes were standardized using the expression levels of *EF-1α* genes. The horizontal coordinate indicates the change of time point, and the vertical coordinate indicates the fold change of the *V. alginolyticus* experiment group compared with those of the PBS control group.

In order to verify the results of transcriptome analysis, we detected the relative expression changes of 16 immune-related DEGs at three time points using quantitative RT-PCR. The fold change detected by the qPCR of selected DEGs was compared with the fold change detected by RNA-Seq. As shown in Figure 6, the expression trends of the selected genes at each time point were verified by qRT-PCR to be the same as the sequencing results, indicating that the RNA-Seq sequencing results in this study were accurate.

## 4. Discussion

### 4.1. Purposes of Transcriptome Research

In recent years, there have been numerous incidents of oyster summer mortality around the world, causing significant economic losses to the oyster industry [30,31]. *Vibrio* infection is considered a key factor in the high mortality of oysters [32]. *V. alginolyticus* is a widely distributed marine Gram-negative bacterium, which causes oyster disease and even mortality of shellfish and shrimp, resulting in severe economic loss [12,13,14]. When the *C. gigas* was infected with *V. splendidus*, stem-like cells with big nuclei and thin cytoplasm were found in the tubules of gill filaments, where DNA synthesis is active and hemocyte production is exuberant [33]. The histopathology of green mussels infected with *V. alginolyticus* shows hemocytic infiltration, the sloughing of tubular epithelial cells, and hepatopancreas destruction [34]. The gills of bivalves, the primary tissues that filter foreign substances, serve as the first defense barrier. Moreover, the gills, as the main tissue of interaction between organisms and environmental factors, have become a key tissue in the study of organisms’ responses to environmental stresses [35]. Tubules of gill filaments were confirmed to be in the hematopoietic position in *C. gigas*, which produce hemocytes with immune defense capabilities [18]. Our observations reveal that the injection of *V. alginolyticus* led to the shedding of epithelial cilia and nucleolysis of epithelial cells in oyster gills. These findings suggest that the gill tissue of the oyster responded to *Vibrio* infection post-injection (Figure S2). The transcriptome sequencing of oyster gills can contribute to a better understanding of how *C. gigas* adapt to pathogen-rich environments. By focusing on the gills, which play a crucial role in the immune response, we aimed to shed light on the molecular processes underlying the interaction between the oyster and the pathogen.

### 4.2. Functional Enrichment Analysis of Immune-Related GO Terms and KEGG Pathways

In order to gain deeper insights into the immune response of *C. gigas* to *V. alginolyticus* infection, we conducted GO and KEGG enrichment analyses using all 1956 DEGs at 12 and 48 h post-injection. The GO enrichment results reveal significant enrichments in immune-related terms, such as the positive regulation of inflammatory response and response to bacterium. Moreover, the KEGG pathway analysis identified several pathways that were significantly enriched, including the inflammatory mediator regulation of TRP channels, the NF-kappa B signaling pathway, and the PI3K-Akt signaling pathway, all of which are closely associated with immunity. These findings suggest that the oyster’s immune system is activated in response to infection, generating an inflammatory response and recognizing bacterial pathogens, and that a variety of immune-regulatory pathways actively respond to the infection with defenses against *Vibrio*.

### 4.3. Speculation of Hub Genes

In general, proteins are the main catalysts, structural constituents, and signaling and molecular machines in living organisms [36]. Systematic studies of protein interactions are useful for exploring the immune regulation of oyster anti-*Vibrio* processes. To delve into this, we constructed a PPI network utilizing 35 genes from the immune-related KEGG pathway. Notably, the number of interactions among the proteins encoded by our identified DEGs exceeded expectations (Table 2), indicating that these proteins interact with each other to maintain immune defense functions. As shown in Figure 5, nodes with more edges are considered to be hub proteins in the immune response process. The corresponding genes are speculated to be the hub genes. Finally, combined with the PPI and KEGG signal pathway, we obtained 16 speculated hub genes (Table 3) from 35 immune-related DEGs for further study.

### 4.4. Functional Analysis of KEGG Signaling Pathways and Hub Genes

This study aimed to delve into the immune defense mechanism of oysters against *V. alginolyticus* by conducting transcriptome analyses of their gill tissues. Through this analysis, we gained comprehensive insights into the crucial pathways and hub genes involved in regulating oyster immune defenses.

#### 4.4.1. Inflammatory Mediator Regulation of TRP Channels

Transient receptor potential (TRP) channels play a crucial role in orchestrating numerous cellular processes, including cytotoxicity, cell differentiation, and cytokine production. They achieve this by exerting direct effects on intracellular cation levels or indirectly regulating various intracellular pathways. The broad impact of TRP channels highlights their significance in cellular functioning and underscores their potential for critical immune defense functions [37,38]. TRP channels generate responses to endogenous factors and messengers during tissue damage and the development of inflammation. It can be activated by many stimuli in the environment, such as temperature changes, chemicals, and pathogenic bacteria [39]. Alterations in intracellular Ca^2+^ concentration can control inflammation and immune cell function. Moreover, TRP channels act as a cation channel that regulates Ca^2+^ permeation. As a result, TRP channels are thought to play a significant role in modulating immune and inflammatory responses [37]. ROS produced by immune cells are not only antimicrobial agents but also signaling molecules [40,41]. In a study of heat stress in the *C. gigas*, it was found that TRPM2 may have a key role in maintaining the ROS response as well as apoptosis in the *C. gigas* [42]. Mollusks can also regulate their own immune responses by activating Trpm2 channels [43]. PTGER4 is an important receptor that can be used to detect a variety of physiological and pathological stimuli as well as orchestrate various biological processes, including inflammatory responses, apoptosis and cytokine production [44,45,46]. Previous studies have shown that PTGER4 transcript levels are significantly upregulated in oyster gills following pathogen attack [44], which is the same as our result at the onset of infection, further suggesting a role in the oyster’s defense response to bacterial pathogens.

#### 4.4.2. NF-κB Signaling Pathway

Nuclear factor-κB (NF-κB) refers to a family of transcription factors that collectively regulate the expression patterns of numerous genes involved in inflammation and cell proliferation [47,48]. NF-κB is found in nearly all animal cells, and it plays a crucial role in cellular responses to various external stimuli, including cytokines, radiation, heavy metals, and bacterial infections [49]. The NF-κB signaling pathway is particularly important in regulating inflammation, immune responses, and stress responses [50]. In our study, we found that the hub genes *BIRC2*, *BIRC3*, and *LBP* are enriched in the NF-κB signaling pathway. Apoptosis is a fundamental biological process that regulates cellular biological growth, development and immune response [51]. BIRC2 and BIRC3 belong to the family of inhibitors of apoptosis (IAP), playing a crucial role in the regulation of NF-κB signaling and apoptosis. These proteins are involved in the intricate balance between cell survival and programmed cell death, exerting their influence on key cellular processes [52]. They promote ubiquitination to regulate innate immunity and inflammatory responses [53]. During pathogen infection, BIRC2 and BIRC3 inhibit apoptosis by ubiquitination and promoting proteasomal degradation [54]. The overexpression of *BIRC2* in zebrafish larvae promoted the proliferation of *Edwardsiella piscicida*, leading to decreased larvae survival [53]. In the present study, *BIRC2* was consistently inhibited in oysters infected with *Vibrio*. However, *BIRC3* was overexpressed in the early stage of infection (12 h) and repressed in the late stage of infection (48 h). *LBP* encodes the lipopolysaccharide-binding protein that plays an essential part in both innate immunity against bacterial infections and will protect the host from Gram-negative bacteria [55]. *V. alginolyticus* is classified as a Gram-negative bacterium, characterized by the presence of a lipopolysaccharide (LPS) on its outer cell wall [56]. LPS is an important microbial surface pathogen-associated molecular pattern of *V. alginolyticus*, which is recognized by the pattern recognition receptor LBP to initiate innate immunity [57]. In the resistance assay of *Trachinotus ovatus* to bacterial infection, LBP exhibited antibacterial and binding activity against Gram-negative bacteria. LBP expression was significantly elevated after *V. alginolyticus* stimulation, enhancing the resistance of *Trachinotus ovatus* to *V. alginolyticus* infection [55]. In our study, LBP was consistently overexpressed in *C. gigas* stimulated by *V. alginolyticus*, which may enhance oyster resistance to *Vibrio*.

#### 4.4.3. The Top Three Key Genes in Terms of Number of Interactions

In this study, we screened for important genes based on the interaction relationships between proteins. After subjecting *C. gigas* to stress induced by *V. alginolyticus*, the key genes that emerged as having the highest number of interactions and potentially influencing the regulation of *C. gigas* immunity were identified as *CASP3*, *MET*, and *PIK3CA*. These hub genes hold significant importance in understanding the immune response of *C. gigas* to *V. alginolyticus* stress. Apoptosis is an important process that prevents tissue damage by removing damaged cells, and is an important means of host clearance of pathogens [58]. Apoptosis is also a key immune defense mechanism against viral, parasitic and bacterial infections in oysters [59,60]. *CASP3* is responsible for encoding the production of caspase-3 protein, a crucial member of the caspase enzyme family. Caspase-3 plays a pivotal role in regulating both apoptosis and inflammatory responses within the cell [61,62]. In others’ studies, the expression of *CASP3* in oysters infected with OsHV-1 showed an upregulation and then a decrease with the time of infection [63], which is the same as our study. MET, a transmembrane tyrosine kinase receptor, is known to bind to hepatocyte growth factors (HGF) and regulate various cellular immune functions, including anti-inflammatory activity, cytokine production, cell migration, and adhesion [64,65]. Gills are recognized as the primary organ of interaction between the organism and the environment, and various pollutants in seawater are filtered through the gills and into the oyster [66]. As a nuclear factor, phosphoinositide 3 kinase (PI3K) can affect a diverse array of cellular functions, encompassing immunity, growth, survival, and cell signaling pathways [67]. Notably, previous studies have elucidated the role of PI3K in regulating immune responses against pathogens and environmental contaminants, while also coordinating phagocytosis in mollusks [68]. Within the PI3K pathway, PIK3CA serves as a crucial regulatory and catalytic subunit, activating PI3Ks in response to extracellular stimuli. This highlights the pivotal involvement of PIK3CA in orchestrating the activation of PI3Ks in various cellular contexts [69]. Previous studies have provided evidence that PIK3CA is involved in regulating various immune response processes, including inflammatory responses and immune cell activation. The upregulated expression of *PIK3CA* improves *Scophthalmus maximus* resistance to *Vibrio anguillarum* and may play a potential role in the innate immune response of organisms to pathogen invasion [70]. Building upon these findings and our previous research [28], our current study reveals that the PI3K-AKT signaling pathway is activated in both blood and gill tissues of oysters as a defense mechanism against *V. alginolyticus* invasion. This highlights the crucial role of PIK3CA and the associated signaling pathway in orchestrating the immune response in oysters.

#### 4.4.4. Other Important Hub Genes

In addition to our main findings, we also discovered several other hub genes that play crucial roles in the immunoregulatory process during *V. alginolyticus* infection. These hub genes include *ARF1*, *CDC42*, *ITGA9 PLG*, and *SESN1*. ARF1, a member of the ARFs family, regulates intracellular vesicle transport and participates in intracellular signaling [71]. The expression of ARF1 was upregulated in vivo when *Amphioctopus fangsiao* was subjected to pathogen stress, affecting humoral immunity and thus regulating the in vivo immune process [72]. CDC42 acts as a regulatory factor to control the production of functional granulocytes in the oyster, thereby regulating the phagocytosis of hemocytes [73]. ITGA9 is a subunit of integrin, a cell surface receptor that plays a crucial role in promoting cell migration and regulating various cellular biological functions, including tumor cell proliferation, adhesion, and invasion [74]. PLG is a key regulatory protein that both promotes fibrinolysis during the wound healing process and aids in rapid wound healing, as well as neutrophil apoptosis and efferocytosis to reduce inflammation [75]. SESN1, a stress-inducible protein, exerts significant cytoprotective functions during a variety of cellular stresses [76]. At this stage, the function of these genes in invertebrates is still understudied, so the genes involved in the immune process after gill infection with *V. alginolyticus* in the oyster need to be further investigated to reveal their specific functions.

## 5. Conclusions

In this study, we aimed to unravel the immune-regulatory mechanisms involved in the infection of *C. gigas* with *V. alginolyticus* by conducting transcriptome analysis of the oyster gills. Our findings, supported by KEGG and PPI results, highlight the significance of several key components in the immune response of *C. gigas*, including the NF-κB signaling pathway, TRP channels, *CASP3*, *MET*, and *PIK3CA*. These results deepen our understanding of the immune mechanisms of oysters against bacteria and provide a rich genetic resource for future studies of oyster immune responses to *V. alginolyticus* infections, which will help to breed oysters with greater resistance to bacteria.

## Figures and Tables

**Table 1:** Summary of 12 immune-related KEGG pathways.

Pathways	Number of DEGs
Inflammatory mediator regulation of TRP channels	7
Central carbon metabolism in cancer	5
PI3K-Akt signaling pathway	5
Salmonella infection	3
NF-kappa B signaling pathway	3
MicroRNAs in cancer	3
Pathways in cancer	3
Endocytosis	2
Staphylococcus aureus infection	2
p53 signaling pathway	2
Apoptosis	2
C-type lectin receptor signaling pathway	2

**Table 2:** Network statistics of immune-related proteins.

Network Stats	
Number of nodes	27
Number of edges	145
Average node degree	10.7
Clustering coefficient	0.709
Expected number of edges	99
PPI enrichment *p*-value	8.29 × 10^−6^

**Table 3:** Summary of 16 key DEGs.

Gene Name (Abbreviation)	Gene Name (Official Full Name)	Number of Protein–Protein Interactions	Number of KEGG Signaling Pathway
CASP3	Caspase 3	20	3
MET	MET proto-oncogene, receptor tyrosine kinase	19	1
PIK3CA	Phosphatidylinositol-4,5-bisphosphate 3-kinase catalytic subunit alpha	17	4
CDC42	Cell division cycle 42	16	1
PLG	Plasminogen	16	1
EP300	E1A binding protein p300	15	1
ITGA9	Integrin subunit alpha 9	14	1
BIRC3	Baculoviral IAP repeat containing 3	13	1
PTGER4	Prostaglandin E receptor 4	13	2
BIRC2	Baculoviral IAP repeat containing 2	12	1
VWF	Von Willebrand factor	12	1
RET	Ret proto-oncogene	11	2
ARF1	ADP ribosylation factor 1	10	1
SLC7A5	Solute carrier family 7 member 5	9	2
SESN1	Sestrin 1	6	1
LBP	lipopolysaccharide binding protein	4	1

## Data Availability

We have uploaded and published our raw data in the Sequence Read Archive database on NCBI. The SRA accession numbers are SRR27190592, SRR27190593, SRR27190594, SRR27190595, SRR27190596, SRR27190597, SRR27190598, SRR27190609, SRR27190615, SRR27190616, SRR27190617, SRR27190618, SRR27190619, SRR27190620, and SRR27190621. The specific website for querying these data is: https://www.ncbi.nlm.nih.gov/Traces/study/?acc=PRJNA861156&o=library_name_s%3Aa%3Bacc_s%3Aa (accessed on 12 December 2023).

## Supplementary Materials

The following supporting information can be downloaded at: https://www.mdpi.com/article/10.3390/ani14111707/s1, Figure S1: The correlation coefficients between the transcriptome data of the three biological replicates; Figure S2: Histological sections of gills of *C. gigas* pre- and post-injection with *V. alginolyticus*. Arrowheads represent epithelial cilia of gills; Table S1: The quality of RNA; Table S2: Information on primers used for quantitative validation of RT-PCR; Table S3: Sequencing results; Table S4: All DEGs; Table S5: GO result.
