# Supplementary material for: Exploration of Molecular Mechanisms of Immunity in the Pacific Oyster (Crassostrea gigas) in Response to Vibrio alginolyticus Invasion
Source: Animals (Basel). 2024 Jun 6;14(11):1707. doi: 10.3390/ani14111707 (PMC11171025; doi:10.3390/ani14111707)
Supplement: Supplementary file 1 [file animals-14-01707-s001.zip › Legends of Fig. S1 and Fig. S2.pdf]

Fig. S1. The correlation coefficients between the transcriptome data of the three biological replicates.

Fig. S2. Histological sections of gills of *C. gigas* pre- and post-injection with *V. alginolyticus*. Arrowheads represent epithelial cilia of gills.
