# Supplementary material for: Exploration of Molecular Mechanisms of Immunity in the Pacific Oyster (Crassostrea gigas) in Response to Vibrio alginolyticus Invasion
Source: Animals (Basel). 2024 Jun 6;14(11):1707. doi: 10.3390/ani14111707 (PMC11171025; doi:10.3390/ani14111707)
Supplement: Supplementary file 1 [file animals-14-01707-s001.zip › Table S2 Information on primers used for quantitative validation of RT-PCR.pdf]

Table S2 Information on primers used for quantitative validation of RT-PCR

Gene name	Forward primer (5'-3')	TM(°C)	Reverse primer (5'-3')	TM(°C)	Amplicon length (bp)
ARF1	TGACAGTAACGATTGGGACAGAATG	57	AGAGCTATGGCGACAGGTACAC	59	99
BIRC2	CCGAAGACAGTGAAGTGACAAGAG	58	TTCATCTGCTGCGTCAATCAAGG	58	92
BIRC3	ACTCGGCAATCAACCAATCATCC	59	CTCCAGACGAACCAGTTCTACATTC	58	114
CASP3	GCCTGTCGTGGGTCGTCTC	61	CCTGGTGTGGTTGCGTAAGC	60	137
CDC42	ATCAAGTCAGAACGCAGGAATACAG	60	AGAAGTCACGCACGCTCTCC	58	96
EP300	CTGAGGTCTCCAATGGCGAATAC	58	CGGTGGCTCCTGCGGTAAG	62	127
ITGA9	CGTTACGCCGAGTTCTCAATGG	59	ACCACGCCTCCTGACCAATC	60	113
LBP	AAGGCGGAAACGGATTGCTCTG	61	TCTGTCCGTCTGTCCGTCTGTC	61	106
MET	GCGACACGAAGACGGTATATTGAG	58	GAGGACTACTCCATAGGACCACAC	59	117
PIK3CA	GGTGGAACGAGTGGCTGGAG	61	TGACAGAGCAGATGGACAGACAG	59	81
PLG	TGACGCCAGCCTTGATGTTAATAG	58	CCACTTCTGACGAGCACATAACC	58	144
PTGER4	CCTGACGATGAAACTGGCTAAGAC	58	TGATGGGCAGCATTACGAACTAAC	58	117
RET	CTTCCTCTACAGCCCAGTCTCAG	59	GACAATGGAGCCGACGCAATC	59	144
SESN1	CGACTCGGACTATACCAGTGACAG	59	TCTTCAGTTGGCTCCTCCTCTTG	56	123
SLC7A5	CAGCGTTCGTGGAGACCTTG	59	CAACAGCAGGAATACACAGATGAAG	58	146
VWF	ATTGGCGATTTGGCGGGAAG	59	CGAAGCGGAGTAGTAGTAGTCATTG	57	120
EF-1α	AGTCACCAAGGCTGCACAGAAAG	60	TCCGACGTATTTCTTTGCGATGT	58	200
GAPDH	TTCTCTTGCCCCTCTTGC	57	CGCCCAATCCTTGTTGCTT	57	127
RO21	AATGCCAGGCTAACAGACCACA	59	TTGGATTTCTGAGATTCCGATCTTC	57	100
RS18	GCCATCAAGGGTATCGGTAGAC	58	CTGCCTGTTAAGGAACCAGTCAG	58	168
RL7	TCCCAAGCCAAGGAAGGTTATGC	60	CAAAGCGTCCAAGGTGTTTCTCAA	58	242
