# Supplementary material for: Exploration of Molecular Mechanisms of Immunity in the Pacific Oyster (Crassostrea gigas) in Response to Vibrio alginolyticus Invasion
Source: Animals (Basel). 2024 Jun 6;14(11):1707. doi: 10.3390/ani14111707 (PMC11171025; doi:10.3390/ani14111707)
Supplement: Supplementary file 1 [file animals-14-01707-s001.zip › Table S3 Sequencing results.pdf]

Table S3. Sequencing results.

Time points	Read length (bp)	Raw reads	Clean reads	Clean Q20 (%)	Clean Q30 (%)	GC (%)	Mapped reads ratio (%)	Detected gene number
BCG-0 h-1	150	46,513,974	33,703,287	97.35	92.7	41.65	72.46	24898
BCG-0 h-2	150	48,663,920	35,511,772	97.47	92.93	41.75	72.97	24866
BCG-0 h-3	150	44,333,618	34,171,080	97.52	93.11	44.22	77.08	24721
PCG-12 h-1	150	50,834,824	37,654,071	97.27	92.43	41.92	74.07	25547
PCG-12 h-2	150	47,003,464	35,306,726	97.58	93.15	42.78	75.12	24769
PCG-12 h-3	150	43,345,228	34,098,018	97.68	93.49	44.92	78.67	24831
VEG-12 h-1	150	45,743,876	34,105,854	97.63	93.36	42.35	74.56	24800
VEG-12 h-2	150	50,441,838	36,644,434	97.27	92.51	41.72	72.65	24895
VEG-12 h-3	150	44,632,000	33,787,684	97.21	92.37	43.83	75.70	24760
PCG-48 h-1	150	47,157,954	33,677,121	97.29	92.35	41.33	71.41	25634
PCG-48 h-2	150	53,137,540	39,125,063	97.25	92.51	42.07	73.63	25895
PCG-48 h-3	150	46,119,000	35,371,498	97.47	92.94	43.27	76.70	25266
VEG-48 h-1	150	45,597,772	35,347,029	97.77	93.64	44.19	77.52	24638
VEG-48 h-2	150	45,121,954	33,839,744	97.57	93.16	42.66	75.00	24589
VEG-48 h-3	150	44,871,196	35,048,145	97.74	93.59	44.14	78.11	25038
