# Supplementary figures and images for: Exploration of Molecular Mechanisms of Immunity in the Pacific Oyster (Crassostrea gigas) in Response to Vibrio alginolyticus Invasion
Source: Animals (Basel). 2024 Jun 6;14(11):1707. doi: 10.3390/ani14111707 (PMC11171025; doi:10.3390/ani14111707)

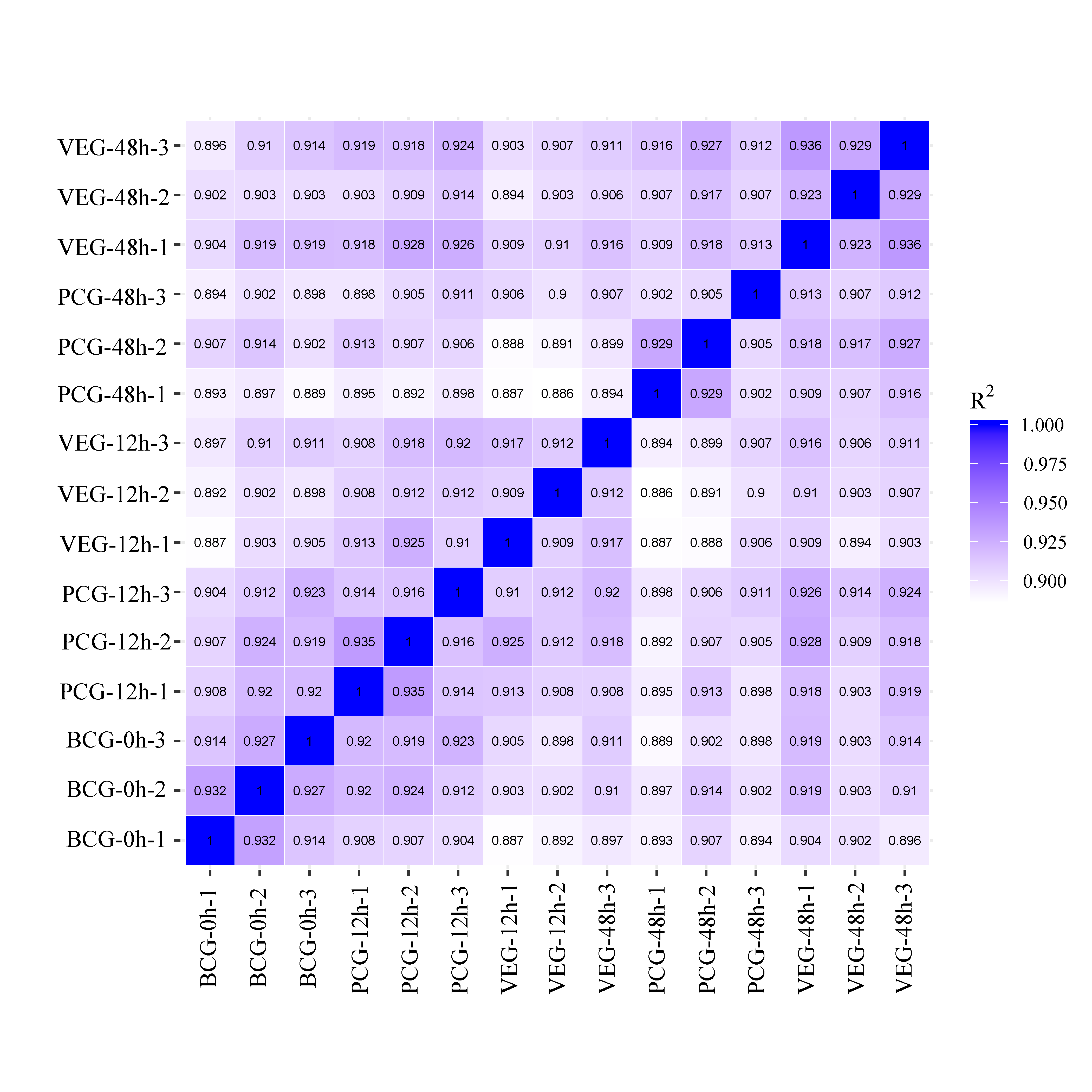

Supplement: Supplementary file 1 [file animals-14-01707-s001.zip › Fig. S1 correlation analysis.png]

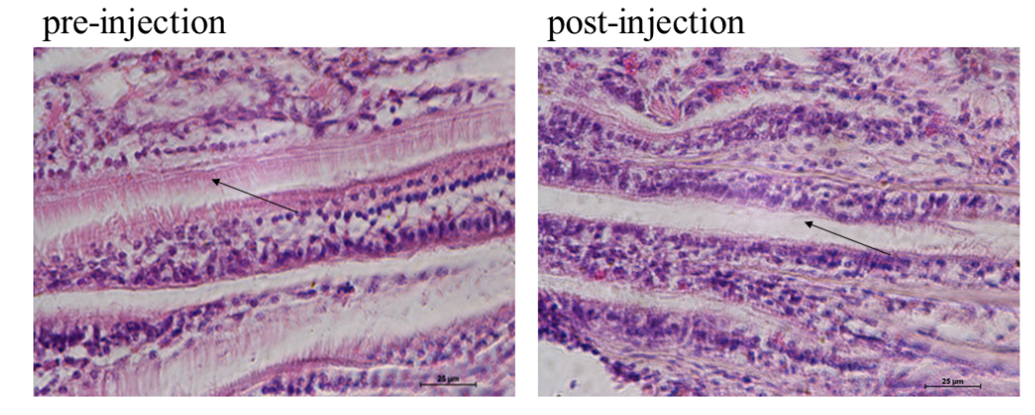

Supplement: Supplementary file 1 [file animals-14-01707-s001.zip › Fig. S2 histological examinations.png]
